# Improved strategy for phylogenetic analysis of classical swine fever virus based on full-length E2 encoding sequences

**DOI:** 10.1186/1297-9716-43-50

**Published:** 2012-06-07

**Authors:** Alexander Postel, Stefanie Schmeiser, Jennifer Bernau, Alexandra Meindl-Boehmer, Gediminas Pridotkas, Zuzana Dirbakova, Miroslav Mojzis, Paul Becher

**Affiliations:** 1 EU and OIE Reference Laboratory for Classical Swine Fever, Institute of Virology, Department of Infectious Diseases, University of Veterinary Medicine, Hannover, Germany; 2 National Food and Veterinary Risk Assessment Institute, Vilnius, Lithuania; 3 Lithuanian University of Health Sciences, Kaunas, Lithuania; 4 State Veterinary Institute Zvolen, Zvolen, Slovak Republic

## Abstract

Molecular epidemiology has proven to be an essential tool in the control of classical swine fever (CSF) and its use has significantly increased during the past two decades. Phylogenetic analysis is a prerequisite for virus tracing and thus allows implementing more effective control measures. So far, fragments of the 5´NTR (150 nucleotides, nt) and the E2 gene (190 nt) have frequently been used for phylogenetic analyses. The short sequence lengths represent a limiting factor for differentiation of closely related isolates and also for confidence levels of proposed CSFV groups and subgroups. In this study, we used a set of 33 CSFV isolates in order to determine the nucleotide sequences of a 3508–3510 nt region within the 5´ terminal third of the viral genome. Including 22 additional sequences from GenBank database different regions of the genome, comprising the formerly used short 5´NTR and E2 fragments as well as the genomic regions encoding the individual viral proteins N^pro^, C, E^rns^, E1, and E2, were compared with respect to variability and suitability for phylogenetic analysis. Full-length E2 encoding sequences (1119 nt) proved to be most suitable for reliable and statistically significant phylogeny and analyses revealed results as good as obtained with the much longer entire 5´NTR-E2 sequences. This strategy is therefore recommended by the EU and OIE Reference Laboratory for CSF as it provides a solid and improved basis for CSFV molecular epidemiology. Finally, the power of this method is illustrated by the phylogenetic analysis of closely related CSFV isolates from a recent outbreak in Lithuania.

## Introduction

Classical swine fever is a devastating animal disease of great economic concern worldwide [1]. The causative agent, classical swine fever virus (CSFV), is highly contagious and infects domestic pigs as well as wild boar. Infection is transmitted either by direct or indirect contact between infected pigs, by contaminated food or swill feeding, but also by transmission *via* contaminated objects and/or persons. Molecular virus tracing helps to understand sources and pathways of infection and therefore is an important tool for disease control [2,3].

During the past two decades technical developments like real-time RT-PCR promoted a reliable and rapid CSF diagnosis [4-6]. CSFV can be divided into three genotypes (1, 2 and 3), each comprising three or four subgenotypes (1.1-1.3; 2.1-2.3; 3.1-3.4) [7,8]. To assign a newly identified CSFV isolate to a genotype and to describe its phylogenetic relations to other known isolates, nucleotide sequencing is mandatory as other techniques like restriction enzyme analysis may allow segregation on genotype level, but resolution on subgenotype level is often insufficient [7,9-11]. Within the highly variable genus *Pestivirus* (single stranded positive-sense RNA viruses) CSFV is the least variable member [7]. During long lasting epidemics, CSFV has been shown to be relatively stable and only few nucleotide changes can be expected [7,12,13]. For example, during an outbreak in the Netherlands in 1997–1998 sixteen CSFV isolates were genetically characterized. In a time span of more than one year only 0–3 differing nucleotides were observed in the variable E1/E2 encoding region (850 nt) and no nucleotide exchanges were found in a 321 nt fragment located in the 5´NTR [13]. Substitution rates between 2 × 10^-3^ and 5 × 10^-4^ substitutions/nucleotide/year were estimated for different regions of the CSFV genome [7,14,15]. These examples emphasize that a region of high variability and adequate length is required for reliable phylogenetic analyses and molecular epidemiological investigations.

Until now, genetic typing mainly relies on a short fragment (150 nucleotides) of the 5´ non translated region (NTR) [7,8,16]. Furthermore, two additional genome fragments of 190 nt and 409 nt length, located in the E2 [7] and NS5B [17] coding regions, respectively, were proposed for a standardized and harmonized strategy for genetic characterization of CSFV [8]. For this reason, sequences of these three regions were included in the CSFV database (CSFV-DB) of the EU Reference Laboratory for CSF (EURL) in Hannover [18]. To date (January 1^st^, 2012), this web based database provides 662 5´NTR fragment, 526 E2 fragment, and 44 NS5B fragment sequences originating from 927 different CSFV isolates. Furthermore, 592 reference sequences from GenBank are included in CSFV-DB, resulting in a total of 1519 sequence entries. The CSFV collection at the EURL and its corresponding sequence database became a valuable tool for CSFV control in Europe.

In today’s routine diagnostic, genetic typing of CSFV relies on sequences of the 5´NTR and E2 fragments to characterize individual virus isolates. However, the short sequence lengths of these fragments often hamper the ability to distinguish closely related isolates during an outbreak situation and result in phylogenetic analyses showing only low statistical significance as reflected by bootstrap values below 70% [8]. These limitations are the reason for an ongoing debate on how to best improve the strategy for molecular characterization, phylogenetic analysis and classification of CSFV isolates into defined subgenotypes.

New technologies like high throughput sequencing allow rapid determination of whole CSFV genome sequences, but are still cost extensive and only available in a few institutions. Therefore their usage is limited to special scenarios [12]. To achieve broad acceptance, a new strategy should improve the quality of generated data while still being easily practicable, robust and having a good cost-benefit relation.

The rapidly growing number of full-length E2 (1119 nt) encoding sequences in public databases like GenBank and recent publications reflect the interest in this genomic region [19-21]. In addition to the phylogenetic aspect, the E2 coding sequence is of particular interest as the E2 protein is the major immunogen besides the E^rns^ and NS3 proteins [20,22]. For that reason E2 and E^rns^ are suitable targets for diagnostic purposes, including development and implementation of a DIVA (differentiating infected from vaccinated animals) strategy in connection with a live-attenuated marker vaccine [23,24]. Full-length E2 gene sequencing may extend the knowledge about conserved epitopes in the E2 protein suitable for the development of reliable diagnostic tools.

The aim of the present study was to establish an improved strategy for genetic typing of CSFV isolates. The results of our work demonstrate that phylogenetic analyses of either 5´NTR-E2 or full-length E2 encoding sequences allow a clear assignment of CSFV isolates to a subgenotype, being supported by reliable bootstrap values. Discrimination of highly similar virus isolates which were not distinguishable by the analysis of the previously used short, partial 5´NTR or E2 fragment sequences is also possible. Compared to the latter, the analysis of full-length E2 encoding sequences provides a considerable increase of information without requiring more time or higher expenses and is therefore recommended to assist future epidemiological studies on CSF.

## Materials and methods

### CSFV isolates and sequences

All isolates (*n* = 33) selected from the CSFV-DB held at the EURL in Hannover are shown in Table 1. The CSFV-DB was built up in the 1990s to collect European CSFV isolates and for that reason mainly contains isolates of genotype 2; this is also reflected in genotype representation of the sequenced isolates (30/33 isolates belonging to genotype 2). The used set of CSFV isolates corresponds to an applied selection from a former study following the aim to choose a representative heterogeneous set out of the isolates available in the CSF-DB [25]. Additional 52 5´NTR-E2 sequences were obtained from the GenBank data library to achieve a dataset that represents a higher variety of genotypes and subgenotypes. 22 of these 52 sequences were included in the phylogenetic analyses, comprising sequences of 15 genotype 1 isolates, three Asian genotype 2.1 isolates, two rare genotype 3 isolates, as well as subgenotype 2.3 reference strain “Alfort-Tuebingen” and one recombinant isolate (Table 2). Sequences of genotype 3 isolates remain underrepresented (*n* = 3) in this dataset as there is no information available about 5´NTR-E2 sequences of subgenotypes 3.1, 3.2 and 3.3 isolates. 30 of the 52 sequences from GenBank, comprising 16 sequences of the 1.1 genotype [GenBank: AY663656, HQ380231, HM175885, AF531433, AF326963, AY805221, AF092448, AF091507, U90951, EU490425, CQ867021, AF352565, D49533, Z46258, D49532, AY382481], six sequences of the 2.1 genotype [GenBank: GU592790, AY554397, GQ902941, AY367767, FJ529205, HQ148063] and eight sequences of the 2.3 genotype [GenBank: HQ148062, HQ148061, GU324242, GU233734, GU233733, GU233732, GU233731, FJ265020] were used to analyze sequence variability, but were not included in the phylogenetic analyses to reduce tree sizes.

**Table 1:** CSFV isolates selected from EURL virus database.

**catalogue-no.**^**1**^	**isolate name**	**subgenotype**	**year of isolation**	**country**	**host**^**2**^	**GenBank acc. no.**^**3**^
CSF0002	“Atzbuell”	2.3	1984	Germany	dp	JQ411559
CSF0014		2.2	1989	Germany	dp	JQ411560
CSF0021		2.1	1989	Germany	dp	JQ411561
CSF0073		2.2	1990	Austria	dp	JQ411562
CSF0083	“Rostock I”	2.3	1992	Germany	dp	JQ411563
CSF0104	“Diepholz I”	2.3	1994	Germany	dp	JQ411564
CSF0120		2.3	1994	Austria	wb	JQ411565
CSF0277		2.1	1997	Germany	dp	JQ411566
CSF0283		2.1	1997	The Netherlands	dp	JQ411567
CSF0290		2.3	1995	Poland	dp	JQ411568
CSF0291		2.3	1995	Poland	dp	JQ411569
CSF0306		1.3	1986	Malaysia	dp	JQ411570
CSF0309	“Kanagawa”	3.4	1974	Japan	dp	JQ411571
CSF0372		2.3	1996	Czech Republic	wb	JQ411572
CSF0378		2.2	1994	Czech Republic	dp	JQ411573
CSF0391		2.3	1997	Germany	dp	JQ411574
CSF0410	“Congenital Tremor”	outgroup	1964	Great Britain	dp	JQ411575
CSF0436		2.3	1995	Germany	dp	JQ411576
CSF0485		2.3	1997	Germany	wb	JQ411577
CSF0496		2.3	1982	Germany	dp	JQ411578
CSF0573	“Parma”	2.2	1998	Italy	dp	JQ411579
CSF0600		2.3	1998	Germany	wb	JQ411580
CSF0638	“Spante”	2.3	1998	Germany	wb	JQ411581
CSF0708		2.1	2000	Great Britain	dp	JQ411582
CSF0710		2.3	2000	Slovakia	wb	JQ411583
CSF0729		2.3	2000	Germany	wb	JQ411584
CSF0750	“Castellon”	2.3	2001	Spain	dp	JQ411585
CSF0867		2.3	2006	Croatia	dp	JQ411586
CSF0906	“Bergen”	2.2	unknown	The Netherlands	dp	JQ411587
CSF0947	“Brescia”	1.1	1951	Italy	dp	JQ411588
CSF1027		2.3	2007	Hungary	wb	JQ411589
CSF1032		2.3	2007	Slovakia	wb	JQ411590
CSF1048	“Panevezys”	2.1	2009	Lithuania	dp	JQ411591

^1^ All virus isolates were kindly provided by collaborating institutes, which are named as references in the CSF database and in the respective GenBank entries.

^2^ dp, domestic pig; wb, wild boar.

^3^ 5´NTR-E2 sequences (3508–3510 nt).

**Table 2:** GenBank sequences used for phylogenetic analyses.

**isolate name**	**subgenotype**	**GenBank acc. no.**
“Koslov”	1.1	HM237795
“Alfort187”	1.1	X87939
“Riems”	1.1	AY259122
“Glentorf”	1.1	U45478
“cF114”	1.1	AF333000
“Shimen-HVRI”	1.1	AY775178
“LOM”	1.1	EU789580
“India”	1.1	EU857642
“JL1-06”	1.1	EU497410
“SWH”	1.1	DQ127910
“CAP”	1.1	X96550
“Brescia”	1.1	AF091661
“CS”	1.2	AF099102
“RUCSFPLUM”	1.2	AY578688
“BRESCIAX”	1.2	AY578687
“SXCDK”	2.1	GQ923951
“SXYL2006”	2.1	GQ122383
“0406/CH/01/TWN”	2.1	AY568569
“strain 39”	recombinant	AF407339
“Alfort-Tuebingen”	2.3	J04358
“94.4-IL-94-TWN”	3.4	AY646427
“P97”	3.4	L49347

Furthermore, CSFV positive samples were obtained from a CSF outbreak in Lithuania in 2011. From each of the five pig holdings affected, two samples were chosen for determination of full-length E2 encoding sequences and subsequent phylogenetic analysis. The five cases were connected geographically and in time as they occurred in holdings with a maximum distance of 15 km in between and within a time period of 38 days. The first case (confirmed on June, 1^st^), the second and the third case (June, 3^rd^) were connected directly to each other as the respective pig holdings belonged to the same company. From the first affected holding piglets were delivered to holding no.2 (on May, 10^th^) and holding no.3 (on May, 19^th^). The epidemiological link to the cases no.4 (confirmed on July, 4^th^) and no.5 (July, 8^th^) is unknown, but it was speculated that virus was transmitted by movement between the farms.

### Isolation of viral RNA

All RNAs were isolated from cell culture supernatants derived from cells infected with CSFV isolates of the CSF virus collection located at the EURL, Hannover. RNA was purified from 140 μL supernatant of an infected PK15 cell culture using the ViralAmp RNA purification kit as recommended by the manufacturer (Qiagen, Hilden, Germany). RNA from samples of the recent CSF outbreak in Lithuania in 2011 was isolated from organ and serum samples using the ViralAmp and the RNeasy kit (Qiagen), respectively.

### Reverse transcriptase-polymerase chain reaction (RT-PCR)

Prepared RNA (6 μL) was added to 26 μL of the reaction mix containing 8 μL 5× RT buffer (MLV-RT kit, Invitrogen, Karlsruhe, Germany) and 2.5 mM of each dNTP (Roche, Mannheim, Germany) for pre-incubation (70°C, 5 min; 4°C). Meanwhile, 8 μL RT mastermix (MLV-RT kit, Invitrogen) containing 0.35 pmol DTT, 400 ng random hexamers, 5 U RNase inhibitor and 400 U M-MLV reverse transcriptase were prepared and added to the pre-incubated reaction mix. Thermocycling was performed under following conditions: 22°C, 5 min; 37°C, 15 min; 42°C, 30 min; 99°C, 5 min; 4°C. The obtained cDNA (1 μL) was used as PCR template for amplification with a high fidelity Phusion polymerase (FinnEnzymes, Fisher Scientific, Schwerte, Germany). The reaction mix (50 μL) contained 10 pmol of each dNTP (Roche), 30 pmol of each primer (Sigma-Aldrich, Taufkirchen, Germany), 5 × reaction buffer (Phusion Buffer HF) and 1 U Phusion polymerase. The thermo profile was set up as follows: 98°C, 30 s; 35 × (98°C, 15 s; 54°C, 30 s; 72°C, 50 s); 72°C, 5 min; 4°C.

In case that amounts of generated amplicon were insufficient, a second PCR run with the same pair of primers was performed using a DeepVent proofreading polymerase (New England BioLabs, Frankfurt am Main, Germany). The PCR product from Phusion PCR (8 μL) was added to a mastermix (92 μL) containing 30 pmol of each dNTP (Roche), 100 pmol of each primer, 10 × Thermopol reaction Buffer (New England BioLabs) and 2 U DeepVent Polymerase. The PCR program was set up as follows: 94°C, 30 s; 35 × (94°C, 30 s; 54°C, 30 s; 72°C, 1:45 min); 72°C, 5 min; 4°C.

### Double stranded nucleotide sequencing

A set of CSFV reference isolates (*n* = 33) from the EURL virus database was used to expand the knowledge on sequence variability in the 5´-region of the CSFV genome. A region of 3508–3510 nucleotides including the commonly sequenced 5´NTR fragment, the region encoding for the N-terminal protease N^pro^ and the structural proteins (C, E^rns^, E1 and E2) was amplified by RT-PCR as described above. RT-PCR amplicons were separated by agarose gel electrophoresis (100 V, 30 min) and purified using a commercial kit according to the manufacturer’s recommendations (GeneJet Gel Extraction Kit, Fermentas, St. Leon-Rot, Germany). Band elution from agarose gel resulting in an amount of at least 15 ng DNA/μL was suitable to obtain sequences of good quality and length of 800–1000 nucleotides. Purified amplicons were subjected to double-stranded Sanger sequencing (Qiagen). Broad reacting sequencing primers were designed using sequences available from the GenBank database (Table 2).

### Sequence analysis

Sequences were analyzed, edited and trimmed using the freeware program Gentle (by M. Manske). Multiple Sequence Alignment was performed by the “MUltiple Sequence Comparison by Log- Expectation” tool (MUSCLE) [26]. Different parts of these sequences were used for phylogenetic analysis. Distances of sequences were calculated by the Kimura-2 parameter method [27] and trees were generated by Neighbor Joining using HUSAR 5.0 (DKFZ, Heidelberg, Germany) which provides the GCG [28] and PHYLIP software packages [29-31]. Alternatively, sequence distances were analyzed by the Hasegawa-Kishino-Yano (HKY) substitution model and phylogenetic trees were generated by Maximum Likelihood (PHYLIP) and Bayesian analysis (MrBayes), respectively, using the TOPALI v2.5 software [32]. Bayesian analysis was performed in two runs, for 2 million generations, 1000 samples and 20% burn-in. Trees were rooted at strain Great Britain/1964 “Congenital Tremor” (CSF0410, [GenBank: JQ411575]) and displayed using Dendroscope 3.0 beta [33]. Similarity Blotting and Bootscanning was performed with the genotype 2.1 and 2.2 isolates using the SimPlot v3.5.1 software (RaySoft, John Hopkins University). Variability of nucleotide positions was analyzed by Mega5 [34] using an extended dataset including additional available sequences (*n* = 85).

## Results

### Amplification and determination of CSFV sequences

**Figure 1:**
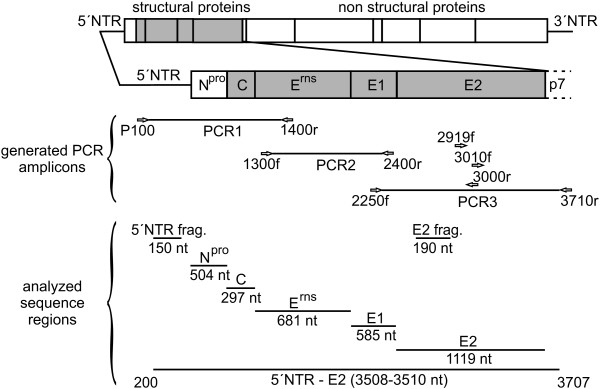
**Strategy for RT-PCR and nucleotide sequencing.** The 5´-terminal portion of the classical swine fever virus genome encompassing parts of the 5´ nontranslated region (NTR) and the region encoding the N-terminal autoprotease N^pro^ and the structural proteins C, E^rns^, E1, and E2 was amplified by RT-PCR in three overlapping amplicons (PCR1, PCR2 and PCR3). The analyzed regions include the commonly used 5´NTR fragment (150 nt) and E2 fragment (190 nt) sequences. Location of primers (indicated by arrows) and sequence length of the 5´NTR-E2 region (3508–3510 nt) correspond to the sequence of CSFV strain Alfort187 [GenBank: X87939] and can differ between the isolates due to presence or absence of nucleotides in the 5´NTR. Further information on all primers is given in Table 3.

The 5´-terminal part of the CSFV genome was amplified in three overlapping amplicons to determine a nucleotide sequence of 3508–3510 nucleotides, comprising a part of the 5´NTR and the genomic region encoding N^pro^, C, E^rns^, E1, E2, and the N-terminal two thirds of p7 (Figure 1). For this purpose, regions conserved among all CSFV genotypes were identified by multiple sequence alignment using 52 CSFV sequences available from GenBank. Three primer pairs for RT-PCR based amplification of the target sequences and two additional sequencing primers located in the E2 coding sequence were designed (Figure 1, Table 3). For all of the 33 isolates included in this study, independently of their genotype, PCR products were obtained (Table 1). PCR amplicons of PCR1, PCR2, and PCR3 showed the expected calculated sizes of 1321 nt, 1136 nt, and 1505 nt, respectively. For each isolate sequences of the three PCR products were determined by double-stranded sequencing and were then assembled to obtain sequences of the 5´NTR-E2 region [GenBank: JQ411559-JQ411591]. GenBank search revealed that the newly determined 5´NTR-E2 sequence of isolate CSF0277 [GenBank: JQ411566] is identical to GenBank entries of two isolates from the same region (“Paderborn”) [GenBank: GQ902941, AY072924]. The sequence of isolate CSF1048 “Panevezys” [GenBank: JQ411591] is identical to the previously published complete genome sequence of this isolate [GenBank: HQ148063].

**Table 3:** Primers used for RT-PCR and nucleotide sequencing.

**primer**	**location**^**1**^	**used for**		**sequence (5´- > 3´)**
		**PCR**	**sequencing**	
P100	99-121	PCR1	+	CATGCCCTTAGTAGGACTAGCAC
CSF1400r	1401-1419	PCR1	+	CACCAYCCRTGTTTRTTCC
CSF1300f	1280-1299	PCR2	+	AAAATATGYAARGGRGTCC
CSF2400r	2396-2415	PCR2	+	AGCCATAYYAYACCTTGCAC
CSF2250f	2224-2243	PCR3	+	TGTTAGRCCRGRYTGGTGGC
CSF3710r	3708-3728	PCR3	+	TRGTYTTRACTGGRTTGTTRG
CSF3000r	2988-3009	-	+	TTYACACATGTCCARTTRCCCC
CSF3010f	2990-3012	-	+	GGYAAYTGGACATGTGTRAAAGG
CSF2919f	2900-2919	-	+	ACCTTCAGGAGAGATAAGCC

^1^ Numbers refer to the sequence of CSFV strain Alfort187 [GenBank: X87939].

### Genetic variability of CSFV

The genetic variability was calculated for all available CSFV sequences (*n* = 85) including the 33 newly determined sequences [GenBank: JQ411559- JQ411591]. Length of the 5´NTR-E2 region differs between 3508 nucleotides and 3510 nucleotides due to one or two additional adenine bases of a poly-adenine stretch located in the 5´NTR. The majority of 5´NTR-E2 sequences (*n* = 62) has a length of 3508 nucleotides, 22 sequences own a length of 3509 nucleotides, and one sequence has a length of 3510 nucleotides [GU233731]. Calculations were performed for the 5´NTR and E2 fragments as well as for the sequences coding for the non-structural protein N^pro^ and the structural proteins to determine the intrinsic discriminatory ability of the individual genomic regions (Table 4). Furthermore, variability was determined for all isolates independently of their genotype or subgenotype assignment (*n* = 85) as well as on genotype level for genotype 2 (*n* = 48) and on subgenotype level for genotype 2.3 (*n* = 28). Genotype 2 and subgenotypes 2.3 were chosen as representatives because they include the majority of the more recently identified CSFV isolates from Europe and reflect the largest number of available sequences. Analysis of the entire 5´NTR-E2 sequences revealed that about 46% of the positions were variable when all genotypes were included, while 34% and 17% variability were observed within genotype 2 and subgenotype 2.3, respectively. With the exception of the more conserved 5´NTR (9% variable nucleotide positions), variability in the different regions encoding the individual proteins was quite uniform (Table 4). The N^pro^ coding sequence and the E2 fragment exhibited a slightly increased variability being about 4-7% higher than the variability of the entire 5´NTR-E2 region (Table 4).

**Table 4:** Genetic variability of different regions within the CSFV genome.

			**number of variable positions**					
**analyzed region**			**all genotypes(*n*** **= 85)**		**genotype 2(*n*** **= 48)**		**subgenotype 2.3(*n*** **= 28)**	
**name**	**position**^**1**^	**size**^**1**^ [nt]	**abs. [n]**	**rel. [%]**	**abs. [n]**	**rel. [%]**	**abs. [n]**	**rel. [%]**
5´NTR frag.	200-349	150	39	26	28	19	14	9
N^pro^	374-877	504	254	50	188	37	101	20
C	878-1174	297	139	47	107	36	52	18
E^rns^	1175-1855	681	309	45	229	34	99	15
E1	1856-2440	585	261	45	179	31	82	14
E2	2441-3361	1119	543	49	424	38	221	20
E2 frag.	2518-2707	190	95	50	78	41	41	21
**5´NTR - E2**	**200-3707**	**3508**	**1621**	**46**	**1202**	**34**	**589**	**17**

^1^ Numbers refer to the sequence of CSFV strain Alfort187 [GenBank: X87939].

### Genetic distances on genotype, subgenotype and isolate level

**Figure 2:**
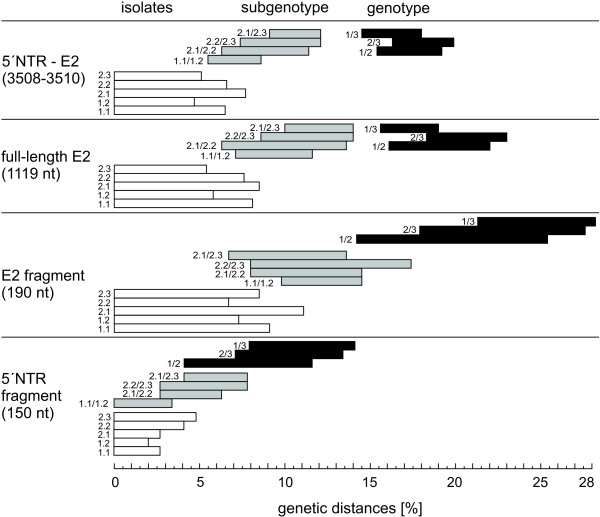
**Ranges of pairwise evolutionary distances among CSFV genotypes, subgenotypes and isolates shown for different genomic regions.** Bars represent minimum and maximum values of genetic distances of the entire 5´NTR-E2 region (3508–3510 nt), the full-length E2 encoding sequence (1119 nt), the E2 fragment (190 nt), and the 5´NTR fragment (150 nt). Genetic distances are shown on isolate level (white bars), on subgenotype level (grey bars), and on genotype level (black bars). The analyzed subgenotypes or pairs of subgenotypes or genotypes are indicated by the numbers preceding the bars. With the exception of CSFV “strain 39” [GenBank: AF407339] and strain Great Britain/1964 “Congenital Tremor” [GenBank: JQ411575], which are not assigned to a defined genotype, all sequences shown in the phylogenetic trees of Figure 3 and Figure 4 are included in the analysis. Genetic distances between sequences were calculated by the Kimura-2 parameter method.

Matrices of genetic distances were generated using the 5´NTR fragment, the E2 fragment, the full-length E2 and the 5´NTR-E2 sequence to find out whether it is possible to establish breakpoints between genotype, subgenotype and isolate level (Figure 2). Genetic distances of longer stretches like the full-length E2 and the 5´NTR-E2 sequences allowed clear segregation between genotypes and subgenotypes, respectively. Genetic distances of the 5´NTR-E2 region varied from ≤ 7.7% among isolates of an individual subgenotype, 5.5%-12.1% between the subgenotypes of one genotype, and 14.5%-19.9% between different genotypes. A similar pattern was found for the full-length E2 encoding sequences displaying evolutionary distances of  ≤  8.5% on isolate level, 6.3%-14% on subgenotype level and 15.6%-23% on genotype level, respectively (Figure 2). This illustrates that even for longer sequence stretches, evolutionary distance between isolates of two different subgenotypes can be higher than the genetic distance between two members of the same subgenotype. Particularly, the observed high variability within genotype 1 as well as between subgenotypes 2.1 and 2.2 does not allow a clear assignment of CSFV isolates to defined/established subgenotypes by the level of genetic distances (Figure 2). A clear segregation can be observed between subgenotypes 2.3 and subgenotypes 2.1 or 2.2, but not between subgenotypes 2.1 and 2.2. To examine whether high similarity of certain sequences within 2.1 and 2.2 might be the result of recombination events, Similarity Plotting and Boot Scanning analysis were performed for isolate CSF0021 (subgenotype 2.1) and isolate The Netherlands/xxxx “Bergen” (CSF0906, subgenotype 2.2) with other representatives of the subgenotypes 2.1 and 2.2. Both isolates exhibit a genetic distance of 6.3% to each other, whereas the genetic distances among individual isolates belonging to subgroup 2.1 was up to 7.7% (isolates “0406/CH/01/TWN” and “SXCDK”). SimPlot analysis of isolate The Netherlands/xxxx “Bergen” showed that the sequence stretch between positions 2941–3509 is more similar to 2.1 sequences than to 2.2 sequences. BootScan was not able to clarify whether this is due to recombination between 2.1 and 2.2 isolates (data not shown).

**Figure 3:**
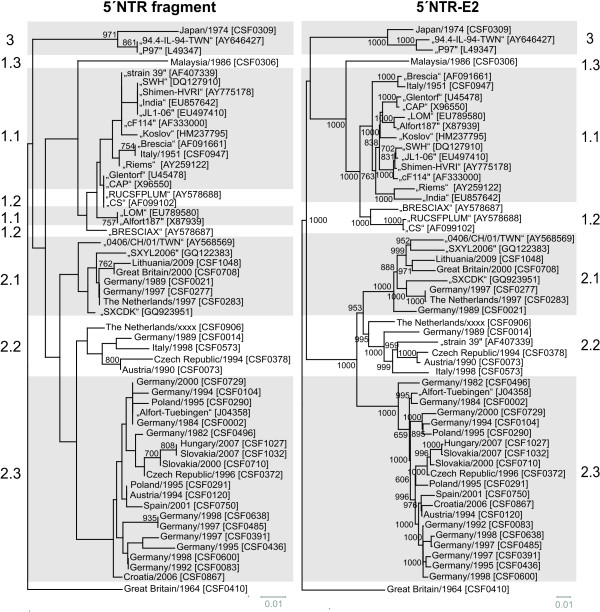
**Phylogenetic trees based on the 5´NTR fragment and the entire 5´NTR-E2 sequences.** Phylogenetic trees of 33 sequences of isolates from the EURL database (country, year, CSF number) and additional 22 reference sequences originating from GenBank (isolate name, accession number) were calculated by the Neighbor Joining method including bootstrap values for 1000 repetitions. Only statistically significant bootstrap values (≥ 70.0%) are indicated. Evolutionary distances between sequences were calculated by the Kimura-2 parameter method. Trees were rooted at the distinct CSFV strain Great Britain/1964 “Congenital Tremor” [GenBank: JQ411575]. Genotypes and subgenotype names are indicated besides the trees [7,8]. Branch lengths are given as 0.01 substitutions per position according to the scale bars underneath each tree.

**Figure 4:**
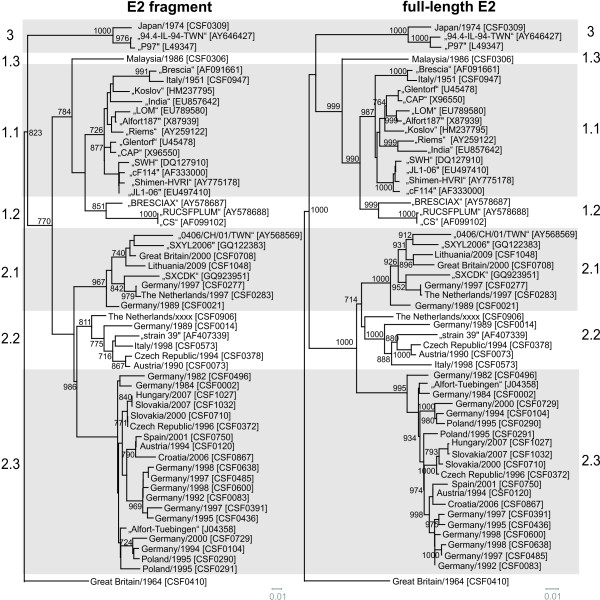
**Phylogenetic trees based on the E2 fragment and full-length E2 encoding sequences.** Phylogenetic trees of 33 sequences of isolates from the EURL database (country, year, CSF number) and additional 22 reference sequences originating from GenBank (isolate name, accession number) were calculated by the Neighbor Joining method including bootstrap values for 1000 repetitions. Only statistically significant bootstrap values (≥ 70.0%) are indicated. Evolutionary distances between sequences were calculated by the Kimura-2 parameter method. Trees were rooted at the distinct CSFV strain Great Britain/1964 “Congenital Tremor” [GenBank: JQ411575]. Genotypes and subgenotype names are indicated besides the trees [7,8]. Branch lengths are given as 0.01 substitutions per position according to the scale bars underneath each tree.

The Chinese CSFV “strain 39” [GenBank: AF407339] is the only naturally emerged recombinant CSFV described so far. “Strain 39” was reported to own a subgenotype 1.1 sequence (strain cF114-like [GenBank: AF333000]) with a replacement of positions 525 and 8398 by a subgenotype 2.1 sequence (GXWZ02-like [GenBank: AY367767]) [35]. Close relatedness between the 5´-terminal portion of “strain 39” and “cF114” (subgenotype 1.1) is confirmed by our analysis of the 5´NTR (Figure 3), whereas the entire NTR-E2 sequence stretch of “strain 39” as well as the E2 fragment and the E2 full-length sequence showed the highest homology with various newly determined sequences of CSFV 2.2 isolates (CSF0014, CSF073, CSF0378, CSF0573, CSF0906) instead of CSFV 2.1 isolates (Figure 3, Figure 4). Genetic distances of these five 2.2 isolates and “strain 39” were 4.7%-8.8% in the full-length E2, whereas the genetic distance between “GWZ02” (subgenotype 2.1) and “strain 39” was 12.7% and thus considerably higher (data not shown). In consequence, the results of our analysis clearly demonstrate that “strain 39” harbors the structural genes of a subgenotype 2.2 isolate rather than of a 2.1 isolate (Figure 3, Figure 4). With the exception of “strain 39”, no other recombination events between different genotypes or subgenotypes could be observed when the trees based on different parts of the 5`NTR-E2 genomic regions were compared.

### Influence of the analyzed region on phylogeny

So far, phylogenetic analysis of CSFV routinely was performed on the basis of the 150 nt 5´NTR fragment and the 190 nt E2 fragment. To analyze the limitations of these short regions and to find a suitable improved strategy, the multiple sequence alignment of the 5´NTR-E2 region (3508-3510 nt) was divided into several subsets, corresponding to the 5´NTR and E2 fragments as well as the regions encoding for the individual viral proteins N^pro^, C, E^rns^, E1, and E2 and subsequently analyzed separately. To achieve better comparability, generated phylogenetic trees were rooted against the most distinct isolate “Congenital Tremor” (CSF0410). With the exception of the 5´NTR, the N^pro^ and the E1 encoding sequences all of the regions resulted in a similar phylogenetic grouping and subgrouping independently of the used method. For these three regions, a clear distinction of isolates of subgenotypes 1.1 and 1.2 was achieved neither by the commonly used Neighbor Joining method nor by other phylogenetic calculations like Maximum Likelihood or Bayesian analysis (data not shown). Neighbor Joining trees of the 5´NTR, the E2 fragment, the full-length E2 and the 5´NTR-E2 sequence rooted at the isolate Great Britain/1964 “Congenital Tremor” (CSF0410) are shown in Figure 3 and Figure 4. Trees based on the E2 fragment and the full-length E2 encoding sequences are similar with the trees applying the complete 5´NTR-E2 sequences. The phylogenetic tree based on the 5´NTR fragment allowed a rough genotyping, but failed to differentiate between the subgenotype 1.1 isolates “CAP” and “Glentorf” and the subgenotype 1.2 strains “CS” and “RUCSFPLUM” (Figure 3, Table 5). Apart from the isolates belonging to genotypes 1.1 and 1.2, the trees based on the N^pro^ and E1 coding sequences showed a relative high resolution (data not shown), whereas in the 5´NTR fragment based tree eleven branches comprised two or more isolates, which were not distinguishable from each other (Figure 3, Table 5).

**Table 5:** **Number of variable positions in individual genomic regions of the CSFV genome**^**1**^**.**

**isolate identification**		**variable nucleotide positions/region**^**2**^							
**name**	**subgenotype**	**NTR frag (150 nt)**	**N**^**pro**^ (504 nt)	**C (297 nt)**	**E**^**rns**^ (681 nt)	**E1 (585 nt)**	**E2 frag (190 nt)**	**E2 (1119 nt)**	**5´NTR-E2 (3508–3510 nt)**
“P97”	3.4	0	16	9	12	12	1	18	75
“94.4-IL-94-TWN”									
“SWH”	1.1	0	31	29	16	37	35	72	196
“JL1-06”									
“Shimen-HVRI”									
“India”									
CSF0947 “Brescia”	1.1	0	5	2	6	2	4	12	27
“Brescia”									
“CAP”	1.1	0	27	13	51	29	21	83	214
“Glentorf”	1.1								
“CS”	1.2								
“RUCSFPLUM”	1.2								
“LOM”	1.1	0	6	1	4	3	3	16	32
“Alfort187”									
CSF0021	2.1	0	29	10	36	19	9	40	138
CSF0277									
CSFO283									
CSF0729	2.3	0	17	11	14	14	10	50	109
CSF0002 “Atzbuell”									
“Alfort-Tuebingen”									
CSF1027	2.3	0	0	0	0	3	0	2	5
CSF1032									
CSF0120	2.3	0	11	8	5	5	6	21	52
CSF0291									
CSF0485	2.3	0	3	1	1	1	1	4	10
CSF0638 “Spante”									
CSF0083 “Rostock I”	2.3	0	3	1	4	1	1	5	14
CSF0600									

^1^ with respect to individual groups of CSFV isolates not distinguishable in the 5´NTR fragment.

^2^ results of ClustalW sequence analysis of 55 CSFV isolates (MUSCLE, v3.8).

To gain more detailed insight into the discriminatory ability of the individual genomic regions, the different sequence data sets subjected to phylogenetic analyses were investigated systematically (Table 5). Some of the individual groups of isolates not distinguishable by the analysis of the 5´NTR fragment comprise strains with an overall high identity reflecting their outbreak history and geographic origin, while other groups encompass strains showing a relatively high sequence divergence with respect to the entire 5´NTR-E2 region (up to 214/3509 variable positions, Table 5). The latter situation was observed for isolates belonging to individual subgenotypes (1.1, 2.1, and 2.3), but also for groups of isolates of different subgenotypes (1.1 and 1.2), again illustrating the limitations of the 5´NTR fragment for discrimination of CSFV isolates. Very closely related and almost identical, recently obtained 2.3 isolates from Slovakia and Hungary (CSF1027, CSF1032), German isolates from the 1990s (CSF0083 and CSF0600; CSF0485 and CSF0638), old subgenotype 1.1 reference strains like “Alfort187” and “LOM” [GenBank: X87939, EU789580] or sequences from two different passages of strain “Brescia” (CSF0947, [GenBank: AF091661]) are either not distinguishable from each other or only at low confidence levels (Figure 3, Table 5).

For most isolates best discrimination was achieved with the sequences encoding for N^pro^ (504 nt) and E2 (1119 nt), respectively (Table 5). Although N^pro^ and E1 coding sequences show a high degree of variability, phylogenetic analyses revealed that these regions are less suited for clear differentiation of 1.1 and 1.2 isolates when compared to analysis of the full-length E2 genes (data not shown). A reliable differentiation of all analyzed strains - even of very closely related isolates – was possible based on phylogenetic analysis of the full-length E2 encoding sequences (Figure 4, Table 5). This is also reflected by significantly higher bootstrap values supporting the clustering in the tree based on full-length E2 gene sequences when compared to phylogenetic analyses based on the E2 fragment (Figure 4). For example, bootstrap values at the 17 nodes within subgenotype 2.3 (≤ 8.5% genetic distance) were significant (≥ 70%) in only five cases when trees were generated with the E2 fragment, whereas 11 and 13 of the 17 nodes showed values ≥ 70% when full-length E2 and the entire 5´NTR-E2 sequences were analyzed, respectively. Accordingly, phylogenetic analysis of the entire 5´NTR-E2 region resulted in only slightly increased bootstrap values when compared to the analysis of full-length E2 encoding sequences, although the former is almost three times longer in size (Figure 3, Figure 4). Taken together, the results of the present study show that phylogenetic analysis of full-length E2 encoding sequences allows differentiation of even closely related isolates and segregation is supported by adequate confidence levels.

### Application of the established strategy during recent Lithuanian CSF outbreak

**Figure 5:**
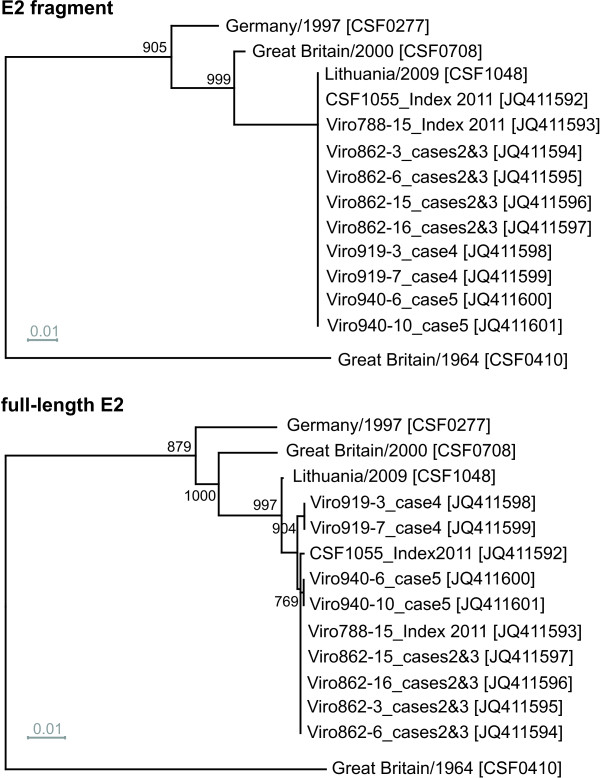
**Phylogenetic analysis of recent CSFV isolates from Lithuania.** The dendrograms were constructed from the E2 fragment (top) and full-length E2 encoding sequences (bottom) of recent Lithuanian CSFV isolates and selected reference strains of genotype 2.1. Full-length E2 encoding sequences of ten Lithuanian isolates obtained during the outbreak in 2011 were deposited in GenBank [GenBank: JQ411592-JQ411601]. For the Lithuanian isolate obtained in 2009 (CSF1048, Panevezys”) the 5´NTR-E2 sequence was determined [GenBank: JQ411591] and found to be identical to a previously published full-genome sequence [GenBank: HQ148063]. Trees were rooted at strain Great Britain/1964 “Congenital Tremor” [GenBank: JQ411575]. Distances were calculated by the Kimura-2 parameter method and used to construct the trees according to the Neighbor joining method. Trees are drawn to scale as indicated by the respective scale bars (0.01 substitutions per position). Bootstrap values were generated by 1000 repetitions; only statistically significant values (≥ 70.0%) are indicated.

In 2011, a CSF outbreak with five involved domestic pig holdings was reported from Lithuania. From each of the five pig holdings affected, two samples were chosen for determination of full-length E2 encoding sequences. At first sight, by routine analysis of the 5´NTR (150 nt) and E2 (190 nt) fragments no sequence differences could be detected to the Lithuanian CSFV isolate originating from an outbreak in 2009. To study the genetic relatedness of these isolates in more detail, the strategy of full-length E2 sequencing and subsequent phylogenetic analysis was applied (Figure 5). All full-length E2 encoding sequences were deposited at GenBank [GenBank: JQ411592-JQ411601]. Comparison of the full-length E2 encoding sequences revealed six and seven nucleotide exchanges between the 2009 sequence and two sequences of samples originating from the index case in 2011. Furthermore, the full-length E2 encoding sequences from four subsequent cases (cases 2–5) were determined for two samples each. The short 5´NTR and E2 fragment sequences displayed no differences between the isolates of the five cases in 2011. In contrast, analysis of the E2 full-length encoding sequences revealed at least three differences between the isolates of case 4 and the isolates from the four other cases in 2011. One of these differing nucleotides was also present in the sequence of the Lithuanian isolate from 2009.

## Discussion

Different regions of the CSFV genome have been proposed for phylogenetic analysis, namely fragments of the 5´NTR as well as partial E2 and NS5B encoding regions [7,8,16,17,36]. During the past two decades, determination of 5´NTR and E2 fragment sequences became the world-wide accepted standard for characterization of CSFV isolates, although this strategy has several limitations which are mainly due to the short sequence lengths of these regions. Today, new technological developments like next-generation sequencing allow rapid determination of full-length sequences, but due to limited access and high expenses the application of such techniques will be restricted to a limited number of institutions and a small number of selected CSFV isolates in the near future. Against this background, rapid and reliable diagnostics in outbreak situations will still rely on analysis of adequate, shorter genomic regions on the basis of an internationally harmonized standard.

To establish an improved strategy for CSFV phylogeny, the 5´NTR-E2 sequences of 33 CSFV isolates from the virus collection held at the EU and OIE Reference Laboratory for CSF (EURL) were determined in this study and used for comparative sequence analyses. For all isolates, including representatives of the three major genotypes, specific amplicons could be generated by RT-PCR using conserved primers. These virus isolates include frequently requested reference strains, isolates of rare CSFV genotypes as well as isolates obtained from recent CSF outbreaks (e.g. in Slovakia, Hungary, Lithuania). It was not possible to include isolates of all known subgenotypes as some subgenotypes (e.g. 3.1, 3.2 and 3.3) are very difficult to obtain and are not represented in the virus collection of the EURL. For most of the sequenced isolates only the short 5´NTR (150 nt) and E2 fragment (190 nt) sequences were available beforehand. Therefore, the 5´NTR-E2 sequences (3508–3510 nt) reported in the present study add significant sequence information to this collection of CSFV isolates. The majority of CSF outbreaks, which occurred during the past decades in Europe, were caused by genotype 2 viruses. In consequence, mainly sequences of genotype 2 virus isolates were determined, comprising 19 isolates of subgenotype 2.3 and five isolates of subgenotypes 2.1 and 2.2 each. Furthermore, 5´NTR-E2 sequences of the two distinct isolates “Congenital Tremor” (CSF0410, no assigned genotype) and “Kanagawa” (CSF0309, genotype 3.4), the reference strain “Brescia” (CSF0947, genotype 1.1) and one Malaysian isolate (CSF0306) of the rare genotype 1.3 were determined.

With regard to the entire 5´NTR-E2 sequences determined in this study and 22 additional sequences obtained from GenBank, all CSFV isolates were assigned to established genotypes and subgenotypes (Figure 3). Our analyses revealed that CSFV “strain 39” [GenBank: AF407339], which has been previously described to be a natural recombinant strain of parental subgenotype 1.1 and 2.1 isolates [35], actually represents a chimera of subgenotype 1.1 and 2.2 isolates (Figure 3, Figure 4). Furthermore, it was recognized that strain The Netherlands/xxxx “Bergen” (CSF0906, subgenotype 2.2) partially displayed a higher genetic similarity to some genotype 2.1 isolates, e.g. to CSFV isolate CSF0021, than to different 2.2 isolates (data not shown). This observation might be a hint for a recombination event between subgenotype 2.1 and 2.2 isolates and is under further investigation. In consequence, strain The Netherlands/xxxx “Bergen” (CSF0906) might disturb segregation of 2.1 and 2.2 isolates when further 2.1 and 2.2 isolates are added in phylogenetic analysis.

Variability and length of analyzed sequences are crucial parameters for the reliability of phylogenetic analyses. The overall variability observed for the different genomic regions is astonishingly uniform (Table 4). Exceptions are the more conserved fragment in the 5´NTR and the slightly more variable E2 fragment. In consequence, not variability but length of the used sequence seems to be crucial to optimize resolution and confidence levels of CSFV phylogeny. Low variability of 9% (14/150 nucleotide positions) in concert with the short sequence length of 150 nt explains the intrinsic limitation of the 5´NTR for phylogenetic analyses. Due to its variability, the 190 nt E2 fragment has the greatest intrinsic discriminatory ability with respect to the above mentioned 5´NTR, E2, and NS5B fragments [7]. The E2 fragment encodes for the N-terminal part of the E2 protein harbouring several neutralizing epitopes resulting in selective pressure [22,37-39]. When comparing the variability of the sequences encoding for the major immunogen E2 and the sequences of other viral proteins like N^pro^, E1 or C, which do not elicit a detectable immune response upon infection, it can be concluded that selection pressure mediated by specific immune reactions is not a major cause of E2 divergence since the overall sequence divergence in other genomic regions reaches similar levels (Table 4). Nevertheless, it can be speculated that lack of antigenic selection pressure might be a reason for the failure of N^pro^- and E1-based analyses to discriminate genotype 1.1 and 1.2 isolates (data not shown). Genotype 1 represents an old and therefore highly variable CSFV genotype. Antigenic selection pressure might have been an important force for development of the 1.1 and 1.2 subgenotypes, while sequence divergence is less pronounced in genomic regions encoding for less immunogenic proteins like N^pro^ and E1. In the present study, analysis of genetic variability in the regions encoding the individual viral proteins (overall 46% variable positions) did not identify regions of adequate length that are more variable than the 504 nt N^pro^ encoding sequence and the 190 nt E2 fragment (50% variable positions). Taking into account the above mentioned limitations of the short 5’NTR fragment as well as the limitations of the nucleotide sequences encoding N^pro^ and E1 for CSFV phylogeny, extension of the short sequence of the E2 fragment to full-length E2 gene sequences is an excellent strategy to obtain data for reliable and detailed phylogenetic analyses (Figure 4).

Calculation and analysis of genetic distances with respect to full-length E2 encoding sequences revealed that genetic distances of more than 15% define a genotype and distances of less than 14% can be found on subgenotype and isolate level (Figure 2). These values will probably not have consistency with an increasing number of analyzed sequences. Furthermore, it was not possible to define universally valid breakpoints between isolate and subgenotype level. Discrimination of the isolate and subgenotype categories based on previously reported ranges for the NS5B fragment (4.5% and 10.5% genetic distance, respectively) is not supported by the analyses of the presented study [8].

For phylogenetic analysis, the use of a standardized method for tree calculation is desirable to achieve a better comparability of internationally published data. In the presented study, genetic distances calculated by the Kimura 2-parameter method and phylogenetic trees generated by Neighbor Joining method subsequently rooted at the strain “Congenital Tremor” (CSF0410) - representing the isolate most distinct from all other CSFV isolates known so far - led to appropriate tree topologies and reliable confidence levels (Figure 3, Figure 4). The phylogenetic trees either generated with full-length E2 encoding sequences or with the 5´NTR-E2 sequences showed the same segregation of CSFV isolates into genotypes and subgenotypes. Compared to E2 full-length sequences, the sequences derived from the 5´NTR and E2 fragments which are currently used for phylogenetic analyses are considerably less suited for differentiation and tracing of CSFV isolates. In case of the 5´NTR fragment the sequence length and intrinsic variability are too low and in case of the E2 fragment the short sequence length significantly limits the information content and consequently diminishes confidence levels of many groupings. The data presented in Figure 3 and Table 5 demonstrate the limited ability of the 5´NTR based trees to differentiate between isolates within a certain subgenotype. In addition, analysis of the 5´NTR fragments fails to segregate isolates into defined subgenotypes as observed for 1.1 and 1.2. This problem was also recognized earlier with other isolates of genotype 1 [7]. Segregation within genotype 1 can be improved by using the E2 fragment, but within a subgenotype, like 2.3, the ability to differentiate closely related isolates (e.g. Slovakian isolates) is still insufficient (Figure 4). Moreover, the trees generated with the E2 fragment sequences display only very low confidence levels which do not allow a further division of the established subgenotypes or a reliable epidemiological interpretation. The high similarity among European isolates, mainly belonging to genotype 2, makes the implementation of a strategy based on larger sequence sets an incontrovertible necessity. This is illustrated by the following examples of CSFV isolates not distinguishable on basis of the short 5´ NTR sequences (Table 5). With respect to the analyzed 5´NTR-E2 sequences, the two isolates CSF0277 (Germany, 1997) and CSF0283 (The Netherlands, 1997) differed in two sites, one of them located in the E2 encoding sequence. These isolates were obtained from a cross-border epidemic and have a direct epidemiological link [40]. Isolates CSF1027 and CSF1032 were obtained from wild boar during the 2007 epidemic in Slovakia and Hungary, respectively, and displayed two nucleotide differences in the E2 encoding sequences. Closely related virus isolates obtained from different German CSF outbreaks in the 1990s (CSF0083 and CSF0600; CSF0485 and CSF0638) were clearly distinguishable on the basis of full-length E2 encoding sequences (Figure 4, Table 5). Furthermore, isolates displaying a high degree of sequence similarity without an epidemiological link (e.g. isolates “LOM” and “Alfort187”) also illustrate the discriminatory ability of the full-length E2 encoding sequences. These examples as well as the recent experiences regarding the Lithuanian outbreaks in 2009 and 2011 clearly demonstrate that the information obtained by analysis of the full-length E2 encoding sequences allows to discriminate even between very closely related virus isolates from the same epidemic and from (nearly) the same geographical origin (Figure 5). Assuming a mutation rate of 3.3 × 10^-3^ to 3.7 × 10^-3^ substitutions/nucleotide/year in the E2 encoding sequence as estimated for the E2 fragment sequence [7,15], approximately 0.6-0.7 nucleotide exchanges may be expected in the short E2 fragment (190 nt) and 3.7-4.1 exchanges in the complete E2 encoding sequence (1119 nt) per year, respectively. Although analysis of full-length E2 encoding sequences results in a significant increase of information, the mutation rate is probably too low for exact determination of infection chains.

To date, both fragments, 5´NTR and E2, are routinely amplified and sequenced for identification and characterization of novel CSFV isolates. The recent CSF outbreak in Lithuania demonstrated that determination of both sequences corresponding to the 5´NTR and E2 fragments was neither able to differentiate between isolates obtained during outbreaks in 2009 and 2011 nor to detect differences between the isolates originating from different outbreak holdings in 2011 (Figure 5). In contrast, phylogenetic analysis of full-length E2 encoding sequences allowed the discrimination of the 2009 and 2011 Lithuanian isolates and identified significant differences between isolates of case no.4 and the isolates of the four other cases. These results suggest that the index case was the source of virus transmission for outbreaks no.2, 3, and 5, while it can be speculated that the virus isolate from case no.4 was introduced either after additional steps of (undetected) transmission or from another source. To allow a reliable interpretation of this finding, more full-length E2 encoding sequences from different CSF epidemics and corresponding epidemiological information need to be analyzed. Against this background, molecular clock analyses of sequences obtained from well documented CSF epidemics would be highly desirable and will be the aim of future studies. Such analyses need to take into account that speed of virus evolution is influenced by many factors including host immunity, vaccination campaigns, presence of virus reservoirs, number of passages in hosts, and last but not least socio-economic determinants [41]. Nevertheless, even without detailed knowledge about speed of molecular evolution in CSF epidemics, the analysis of full-length E2 encoding sequences provides valuable information about the origin of virus introduction as this method increases the probability to identify the ancestral virus isolate. In case of the two Lithuanian outbreaks in 2009 and 2011, identical isolates would have indicated an arrest of molecular clock like in infectious material being frozen (frozen meat, frozen laboratory isolate, etc.). The latter scenario could be clearly excluded by analysis of the full-length E2 encoding sequences. Accordingly, the Lithuanian example illustrates the benefit of phylogenetic analysis of full-length E2 encoding sequences with regard to molecular virus tracing.

Taken together, the proposed strategy based on complete E2 coding sequences allows a clear assignment of CSFV isolates to a subgenotype, results in reliable and statistically significant bootstrap values, and even enables the discrimination of highly similar virus isolates without requiring more time or higher expenses.

## Competing interests

The authors declare that they have no competing interests.

## Authors’ contributions

AP performed and managed RT-PCRs and sequencing, carried out sequence and phylogenetic analyses, contributed to the design of the study and drafted the manuscript. JB performed RNA isolation, RT-PCRs, Gel purification and edited the sequence data. StS and AMB selected, propagated and provided the CSFV isolates, contributed to the design of the study and drafted the manuscript. GP provided Lithuanian CSFV isolates and epidemiological data. ZB and MM characterized and provided Slovakian CSFV isolates and participated in the design of the study. PB designed the RT-PCR primers, evaluated the RT-PCRs, conceived and managed the study, and helped to critically revise the manuscript. All authors read and approved the final manuscript.

## References

[B1] Edwards S Fukusho A Lefevre PC Lipowski A Pejsak Z Roehe P Westergaard J Classical swine fever: the global situation Vet Microbiol 2000 73 103 119 10.1016/S0378-1135(00)00138-3 10785321

[B2] Depner KR Strebelow G Staubach C Kramer M Teuffert J Botcher L Hoffmann B Beer M Greiser-Wilke I Mettenleiter T Case report: the significance of genotyping for the epidemiological tracing of classical swine fever (CSF) Dtsch Tierarztl Wochenschr 2006 113 159 162 16716053

[B3] Moennig V Floegel-Niesmann G Greiser-Wilke I Clinical signs and epidemiology of classical swine fever: a review of new knowledge Vet J 2003 165 11 20 10.1016/S1090-0233(02)00112-0 12618065

[B4] Hoffmann B Beer M Schelp C Schirrmeier H Depner K Validation of a real-time RT-PCR assay for sensitive and specific detection of classical swine fever J Virol Methods 2005 130 36 44 10.1016/j.jviromet.2005.05.030 16055202

[B5] Vilcek S Herring AJ Herring JA Nettleton PF Lowings JP Paton DJ Pestiviruses isolated from pigs, cattle and sheep can be allocated into at least three genogroups using polymerase chain reaction and restriction endonuclease analysis Arch Virol 1994 136 309 323 10.1007/BF01321060 8031236

[B6] Zhao JJ Cheng D Li N Sun Y Shi Z Zhu QH Tu C Tong GZ Qiu HJ Evaluation of a multiplex real-time RT-PCR for quantitative and differential detection of wild-type viruses and C-strain vaccine of Classical swine fever virus Vet Microbiol 2008 126 1 10 10.1016/j.vetmic.2007.04.046 17658704

[B7] Lowings P Ibata G Needham J Paton D Classical swine fever virus diversity and evolution J Gen Virol 1996 77 1311 1321 10.1099/0022-1317-77-6-1311 8683221

[B8] Paton DJ McGoldrick A Greiser-Wilke I Parchariyanon S Song JY Liou PP Stadejek T Lowings JP Bjorklund H Belak S Genetic typing of classical swine fever virus Vet Microbiol 2000 73 137 157 10.1016/S0378-1135(00)00141-3 10785324

[B9] Chen N Li D Yuan X Li X Hu H Zhu B Wan X Fang W Genetic characterization of E2 gene of classical swine fever virus by restriction fragment length polymorphism and phylogenetic analysis Virus Genes 2010 40 389 396 10.1007/s11262-010-0465-8 20217206

[B10] Harding M Lutze-Wallace C Prud'Homme I Zhong X Rola J Reverse transcriptase-PCR assay for detection of hog cholera virus J Clin Microbiol 1994 32 2600 2602 7814509 10.1128/jcm.32.10.2600-2602.1994 PMC264114

[B11] Parchariyanon S Inui K Pinyochon W Damrongwatanapokin S Takahashi E Genetic grouping of classical swine fever virus by restriction fragment length polymorphism of the E2 gene J Virol Methods 2000 87 145 149 10.1016/S0166-0934(00)00162-2 10856761

[B12] Leifer I Hoffmann B Hoper D Bruun Rasmussen T Blome S Strebelow G Horeth-Bontgen D Staubach C Beer M Molecular epidemiology of current classical swine fever virus isolates of wild boar in Germany J Gen Virol 2010 91 2687 2697 10.1099/vir.0.023200-0 20660149

[B13] Widjojoatmodjo MN van Gennip HG de Smit AJ Moormann RJ Comparative sequence analysis of classical swine fever virus isolates from the epizootic in The Netherlands in 1997–1998 Vet Microbiol 1999 66 291 299 10.1016/S0378-1135(99)00017-6 10384890

[B14] Jenkins GM Rambaut A Pybus OG Holmes EC Rates of molecular evolution in RNA viruses: a quantitative phylogenetic analysis J Mol Evol 2002 54 156 165 10.1007/s00239-001-0064-3 11821909

[B15] Zhang H Cao HW Wu ZJ Cui YD Evolutionary rate of E2 genes of classical swine fever virus in China Isr J Vet Med 2011 66 161 163

[B16] Greiser-Wilke I Depner K Fritzemeier J Haas L Moennig V Application of a computer program for genetic typing of classical swine fever virus isolates from Germany J Virol Methods 1998 75 141 150 10.1016/S0166-0934(98)00109-8 9870589

[B17] Bjorklund H Lowings P Stadejek T Vilcek S Greiser-Wilke I Paton D Belak S Phylogenetic comparison and molecular epidemiology of classical swine fever virus Virus Genes 1999 19 189 195 10.1023/A:1008132613228 10595410

[B18] Greiser-Wilke I Zimmermann B Fritzemeier J Floegel G Moennig V Structure and presentation of a World Wide Web database of CSF virus isolates held at the EU reference laboratory Vet Microbiol 2000 73 131 136 10.1016/S0378-1135(00)00140-1 10785323

[B19] Chen N Tong C Li D Wan J Yuan X Li X Peng J Fang W Antigenic analysis of classical swine fever virus E2 glycoprotein using pig antibodies identifies residues contributing to antigenic variation of the vaccine C-strain and group 2 strains circulating in China Virol J 2010 7 378 10.1186/1743-422X-7-378 21194462 PMC3025870

[B20] Wu Z Wang Q Feng Q Liu Y Teng J Yu AC Chen J Correlation of the virulence of CSFV with evolutionary patterns of E2 glycoprotein Front Biosci (Elite Ed) 2010 2 204 220 20036871 10.2741/e83

[B21] Xia H Wahlberg N Qiu HJ Widen F Belak S Liu L Lack of phylogenetic evidence that the Shimen strain is the parental strain of the lapinized Chinese strain (C-strain) vaccine against classical swine fever Arch Virol 2011 156 1041 1044 10.1007/s00705-011-0948-5 21340740

[B22] Peng WP Hou Q Xia ZH Chen D Li N Sun Y Qiu HJ Identification of a conserved linear B-cell epitope at the N-terminus of the E2 glycoprotein of Classical swine fever virus by phage-displayed random peptide library Virus Res 2008 135 267 272 10.1016/j.virusres.2008.04.003 18485511

[B23] Koenig P Lange E Reimann I Beer M CP7_E2alf: a safe and efficient marker vaccine strain for oral immunisation of wild boar against Classical swine fever virus (CSFV) Vaccine 2007 25 3391 3399 10.1016/j.vaccine.2006.12.052 17257713

[B24] Reimann I Depner K Utke K Leifer I Lange E Beer M Characterization of a new chimeric marker vaccine candidate with a mutated antigenic E2-epitope Vet Microbiol 2010 142 45 50 10.1016/j.vetmic.2009.09.042 19892497

[B25] Blome S Grotha I Moennig V Greiser-Wilke I Classical swine fever virus in South-Eastern Europe–retrospective analysis of the disease situation and molecular epidemiology Vet Microbiol 2010 146 276 284 10.1016/j.vetmic.2010.05.035 20541876

[B26] The European Bioinformatics Institute Home Page [http://www.ebi.ac.uk/Tools/msa/muscle/]

[B27] Kimura M A simple method for estimating evolutionary rates of base substitutions through comparative studies of nucleotide sequences J Mol Evol 1980 16 111 120 10.1007/BF01731581 7463489

[B28] Devereux J Haeberli P Smithies O A comprehensive set of sequence analysis programs for the VAX Nucleic Acids Res 1984 12 387 395 10.1093/nar/12.1Part1.387 6546423 PMC321012

[B29] Felsenstein J Confidence limits on phylogenies: an approach using the bootstrap Evolution 1985 39 783 791 10.2307/2408678 28561359

[B30] Felsenstein J PHYLIP (Phylogeny Inference Package) version 3.5c Department of Genetics, University of Washington, Seattle, USA 1993

[B31] Saitou N Nei M The neighbor-joining method: a new method for reconstructing phylogenetic trees Mol Biol Evol 1987 4 406 425 3447015 10.1093/oxfordjournals.molbev.a040454

[B32] Milne I Lindner D Bayer M Husmeier D McGuire G Marshall DF Wright F TOPALi v2: a rich graphical interface for evolutionary analyses of multiple alignments on HPC clusters and multi-core desktops Bioinformatics 2009 25 126 127 10.1093/bioinformatics/btn575 18984599 PMC2638937

[B33] Huson DH Richter DC Rausch C Dezulian T Franz M Rupp R Dendroscope: An interactive viewer for large phylogenetic trees BMC Bioinforma 2007 8 460 10.1186/1471-2105-8-460 PMC2216043 18034891

[B34] Tamura K Peterson D Peterson N Stecher G Nei M Kumar S MEGA5: molecular evolutionary genetics analysis using maximum likelihood, evolutionary distance, and maximum parsimony methods Mol Biol Evol 2011 28 2731 2739 10.1093/molbev/msr121 21546353 PMC3203626

[B35] He CQ Ding NZ Chen JG Li YL Evidence of natural recombination in classical swine fever virus Virus Res 2007 126 179 185 10.1016/j.virusres.2007.02.019 17428567

[B36] Greiser-Wilke I Dreier S Haas L Zimmermann B Genetic typing of classical swine fever viruses--a review Dtsch Tierarztl Wochenschr 2006 113 134 138 (in German) 16716047

[B37] Lin M Lin F Mallory M Clavijo A Deletions of structural glycoprotein E2 of classical swine fever virus strain alfort/187 resolve a linear epitope of monoclonal antibody WH303 and the minimal N-terminal domain essential for binding immunoglobulin G antibodies of a pig hyperimmune serum J Virol 2000 74 11619 11625 10.1128/JVI.74.24.11619-11625.2000 11090160 PMC112443

[B38] van Rijn PA Miedema GK Wensvoort G van Gennip HG Moormann RJ Antigenic structure of envelope glycoprotein E1 of hog cholera virus J Virol 1994 68 3934 3942 7514680 10.1128/jvi.68.6.3934-3942.1994 PMC236899

[B39] van Rijn PA van Gennip RG de Meijer EJ Moormann RJ A preliminary map of epitopes on envelope glycoprotein E1 of HCV strain Brescia Vet Microbiol 1992 33 221 230 10.1016/0378-1135(92)90050-4 1282755

[B40] Greiser-Wilke I Fritzemeier J Koenen F Vanderhallen H Rutili D De Mia GM Romero L Rosell R Sanchez-Vizcaino JM San Gabriel A Molecular epidemiology of a large classical swine fever epidemic in the European Union in 1997–1998 Vet Microbiol 2000 77 17 27 10.1016/S0378-1135(00)00253-4 11042397

[B41] Duffy S Shackelton LA Holmes EC Rates of evolutionary change in viruses: patterns and determinants Nat Rev Genet 2008 9 267 276 18319742 10.1038/nrg2323

